# Integration of Bioinformatics and Machine Learning to Identify CD8+ T Cell-Related Prognostic Signature to Predict Clinical Outcomes and Treatment Response in Breast Cancer Patients

**DOI:** 10.3390/genes15081093

**Published:** 2024-08-19

**Authors:** Baoai Wu, Longpeng Li, Longhui Li, Yinghua Chen, Yue Guan, Jinfeng Zhao

**Affiliations:** 1 Institute of Physical Education and Sport, Shanxi University, Taiyuan 030006, China; wf670720@sxu.edu.cn (B.W.); lp200721@163.com (L.L.); c1846905590@163.com (Y.C.); 18809802068@163.com (Y.G.); 2 Capital University of Physical Education and Sports, Beijing 100191, China; m15235524119@163.com

**Keywords:** breast cancer, machine learning, CD8+ T cell, tumor microenvironment, prognostic signature

## Abstract

The incidence of breast cancer (BC) continues to rise steadily, posing a significant burden on the public health systems of various countries worldwide. As a member of the tumor microenvironment (TME), CD8+ T cells inhibit cancer progression through their protective role. This study aims to investigate the role of CD8+ T cell-related genes (CTRGs) in breast cancer patients. Methods: We assessed the abundance of CD8+ T cells in the TCGA and METABRIC datasets and obtained CTRGs through WGCNA. Subsequently, a prognostic signature (CTR score) was constructed from CTRGs screened by seven machine learning algorithms, and the relationship between the CTR score and TME, immunotherapy, and drug sensitivity was analyzed. Additionally, CTRGs’ expression in different cells within TME was identified through single-cell analysis and spatial transcriptomics. Finally, the expression of CTRGs in clinical tissues was verified via RT-PCR. Results: The CD8+ T cell-related prognostic signature consists of two CTRGs. In the TCGA and METABRIC datasets, the CTR score appeared to be negatively linked to the abundance of CD8+ T cells, and BC patients with higher risk score show a worse prognosis. The low CTR score group exhibits higher immune infiltration levels, closely associated with inhibiting the tumor microenvironment. Compared with the high CTR score group, the low CTR score group shows better responses to chemotherapy and immune checkpoint therapy. Single-cell analysis and spatial transcriptomics reveal the heterogeneity of two CTRGs in different cells. Compared with the adjacent tissues, CD163L1 and KLRB1 mRNA are downregulated in tumor tissues. Conclusions: This study establishes a robust CD8+ T cell-related prognostic signature, providing new insights for predicting the clinical outcomes and treatment responses of breast cancer patients.

## 1. Introduction

Breast cancer is one of the main diseases that threaten women’s health and cause disability and death [1]. Global data from 2020 reveal 2.3 million new cases of breast cancer, representing approximately 12% of all newly diagnosed cancers, along with 685,000 related deaths [2]. With the success of novel systemic therapies, the prognosis of patients with early and advanced breast cancer has steadily and progressively improved [3]. Despite the fact that the majority of early-stage breast cancer cases are curable with various treatment options, the incidence of breast cancer continues to increase [4,5]. Identification of biomarkers is essential for the diagnosis and treatment of breast cancer. Several recent studies have found that circulating tumor cells (CTC), circulating tumor DNA (ctDNA), circulating tumor RNA (ctRNA) and extracellular vesicles (EV) in liquid biopsies have shown great potential as BC biomarkers [6,7,8,9]. As one of the biomarkers, prognostic signature can identify high-risk patients for effective treatment and potentially reduce mortality.

Cancer cells proliferate and survive in complex tissue ecosystems called tumor microenvironments (TMEs), which crucially contribute to cancer cell growth, invasion and metastasis [10]. A diverse range of immune and non-immune cells, as well as many factors secreted by them, are present in the TME, and the interactions of these cells form an intra-tumor environment that is chronically inflammatory, immunosuppressive, and pro-angiogenic [11]. TME plays a key role in dynamically regulating tumor progression and targeting the malignant components of TME shows great potential in the treatment of cancer [12,13]. As part of TME, CD8+ T cells play an important role in inhibiting cancer development. CD8+ T cells have the ability to recognize and kill cancer cells, and tumor cell-specific CD8+ T cells can be measured in tumors [14,15]. CD8+ T cells residing in tumor tissues were found to correlate with the expression of PD-1/PD-L1, which suppresses the immune activity of tumors [16]. In addition, immunotherapies (cytokine therapy, immune checkpoint blockade, chimeric antigen receptor therapy, and overt T cell transfer) that enhance the activity of antigen-specific CD8+ T cells have been successful in many tumors [17,18]. Therefore, constructing CD8+ T cell-related prognostic traits is important for predicting the prognosis of breast cancer as well as the response to treatment.

In this study, we built the CD8+ T cell-related prognostic signature containing two genes by WGCNA and machine learning in different cohorts, and investigated its relationship with immune cell infiltration, immunotherapy and drug sensitivity. These provide important references for predicting clinical outcomes as well as treatment response in breast cancer patients.

## 2. Materials and Methods

### 2.1. Data Acquisition and Processing

Gene expression data and clinical data for TCGA-BRCA, METABRIC, GSE96058, and GSE20685 were obtained from The Cancer Genome Atlas (TCGA) database (https://portal.gdc.cancer.gov/, accessed on 8 January 2024), cBioPortal for Cancer Genomics (https://www.cbioportal.org/, accessed on 8 January 2024), and Gene Expression Omnibus (https://www.ncbi.nlm.nih.gov/geo/, accessed on 15 February 2024). Table S1 shows the baseline characteristics of these datasets. Differential expression genes (DEGs) in the TCGA-BRCA were obtained using the “esayTCGA” R package (Version 0.0.1.7000).

### 2.2. Assessment of CD8+ T Cells Abundance

The abundance of CD8+ T cells was evaluated using four algorithms in the “IBOR” R package (Version 0.99.9), including the Microenvironment Cell Populations-counter (MCP-counter) algorithm, xCell algorithm, quantiseq algorithm, and ssGSEA algorithm.

### 2.3. WGCNA Analysis

Weighted Gene Co-expression Network Analysis (WGCNA) related to CD8+ T cell abundance was conducted using the “WGCNA” R package (Version 1.72-1). We selected the top 5000 genes from the TCGA-BRCA and METABRIC cohorts to construct the network and determined the soft thresholding power. Subsequently, gene modules were identified through clustering, and the relationship between CD8+ T cell abundance and module eigengenes (MEs) was assessed. The gene modules with the most relevance were extracted for further study. Finally, overlapping genes among module genes from TCGA-BRCA, METABRIC, and DEGs were extracted to serve as CD8+ T cell-related genes (CTRGs).

### 2.4. Selection of the Prognostic CTRGs

Prognosis-related CTRGs were screened using the univariate Cox analysis with a screening criterion of *p* < 0.05. These CTRGs were further selected using six machine learning methods, including CoxBoost, random forest algorithm (RFS), extreme gradient boosting (XGBoost), the least absolute shrinkage and selection operator (LASSO), stepwise, and gradient boosting machine (GBM). Eventually, we extracted overlapping genes identified by six machine learning algorithms and further screened genes for constructing prognostic signature by multifactorial Cox.

### 2.5. Construction of the CD8+ T Cell-Related Prognostic Signature

To avoid underfitting of the prognostic model, we used 8 machine learning algorithms to construct the prognostic signature: CTR score. The 8 machine learning algorithms are LASSO, Ridge, SurvReg, StepCox, Survival-Svm, plsRcox GBM, and CoxBoost. To ensure the accuracy of CTR scores, we split the TCGA-BRCA randomly into a training set and a validation set according to a ratio of 6:4. We build the CTR score in the training set and evaluate the performance of the CTR score in the test set. In addition, the METABRIC, GSE96058 and GSE20685 datasets were applied as exterior validation sets to further check the performance of the CTR score. Finally, we selected the highest C-index of the 8 machine learning algorithms to construct the CTR score. The sample in the TGCA cohort was categorized into high and low CTR score groups based on the median CTR score. The impact of CTR score on the overall survival (OS) of BC patients was then assessed through survival analysis. Additionally, ROC curves for CTR score at 1, 3, and 5 years were plotted.

### 2.6. Construction of the Nomogram

The relationship between clinical characteristics and CTR score was analyzed using univariate and multivariate Cox regression analyses. We constructed a nomogram by combining the CTR score with clinical characteristics with independent prognostic value in a multifactorial Cox. Additionally, the ROC curve and calibration curve of the nomogram were plotted.

### 2.7. Functional Enrichment Analysis

Gene Ontology (GO) and Kyoto Encyclopedia of Genomes (KEGG) enrichment analyses were carried out with the ”clusterProfiler” R package (version 4.8.2). Differential biological processes between the various CTR score groups were analyzed by the “GSVA” R package (Version 1.48.0).

### 2.8. Immune Cell Infiltration Analysis

The CIBERSORT, EPIC, TIMER, MCPcounter, quantiseq, and ESTIMATE algorithms were used to measure immune cell infiltration in both high and low CTR score groups. In addition, the correlation of the CTR score with the immunoinhibitor, immunostimulator, major histocompatibility complex (MHC) molecule, chemokine, and chemokine receptor was analyzed. Referring to previous studies [19,20], we analyzed differences in 29 immune signatures and the cancer immune cycle between CTR score groups.

### 2.9. Analysis of Immune Therapy and Drug Sensitivity

Immunophenotype score (IPS) data were downloaded from The Cancer Immunome Atlas (TCIA, https://tcia.at/, accessed on 15 April 2024) and variations in IPS between CTR score groups were assessed. IPS indicates a patient’s sensitivity to the immune checkpoint suppressant. The “oncoPredict” R package (Version 0.2) was used for drug sensitivity analysis of chemotherapy-related drugs, using the half-maximal inhibition concentrations (IC50) to represent drug sensitivity.

### 2.10. Single-Cell and Spatial Transcriptomics Analysis

The expression of CTRGs in different cell types was analyzed using the single-cell database TISCH2 (http://tisch.comp-genomics.org/, accessed on 15 April 2024). Additionally, the spatial distribution of CTRGs was analyzed using the Sparkle database (https://grswsci.top/analyze, accessed on 8 January 2024).

### 2.11. Validation of Prognostic CTRGs Expression Levels

Immunohistochemistry results for CTRGs were downloaded from the Human Protein Atlas (https://www.proteinatlas.org/, accessed on 18 April 2024). Expression data for the CD163L1 protein in breast cancer were obtained from UALCAN (https://ualcan.path.uab.edu/index.html, accessed on 18 April 2024). Expression data for CTRGs in breast cancer cell lines were downloaded from the Cancer Cell Line Encyclopedia (CCLE) (https://depmap.org/portal/, accessed on 22 April 2024), and visualized using the “ggplot2” R package (Version 3.4.2).

### 2.12. Real-Time Fluorescent Polymerase Chain Reaction (RT-PCR)

Clinical tissue from BC patients was provided by the Shanxi Cancer Hospital. The study received permission from the Research Ethics Committee of the Shanxi Cancer Hospital (approval number: KY2023163), and all participants signed informed consent forms. Total RNA was extracted from tissues or cells using the Trizol reagent kit. Reverse transcription was performed using the Takara reagent kit. The real-time fluorescent quantitative PCR steps are as follows: configuration of the reverse transcription system; real-time PCR reaction; calculation of the target gene mRNA expression levels. Primers for two genes are listed in Supplementary Table S2.

### 2.13. Statistical Analysis

The Wilcoxon test for comparing the difference between two groups was used. The Kaplan–Meier analysis was used to evaluate the difference in overall survival (OS) between the low and high CTR score groups. All statistical analyses were performed using R version 4.3.0, with *p* < 0.05 indicating statistical significance.

## 3. Results

### 3.1. Relationship between CD8+ T Cell Infiltration Level and Prognosis

**Figure 1:**
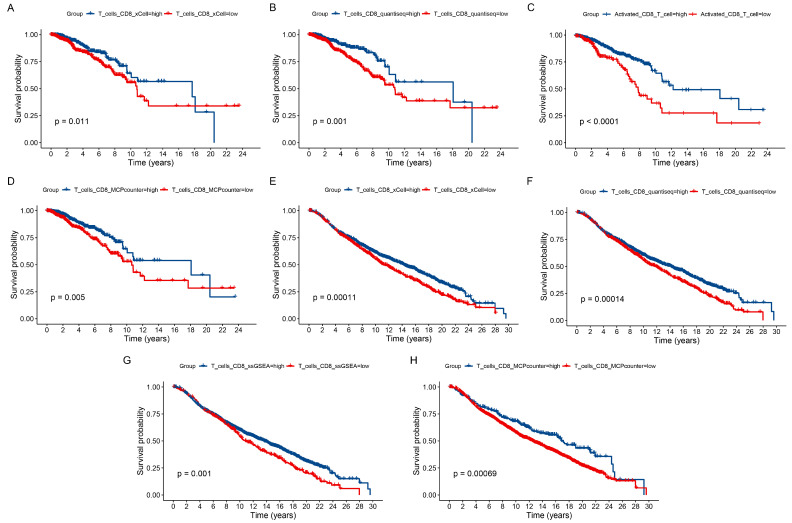
Survival analysis based on CD8+ T cell abundance. Survival curves for different CD8+ T cell abundances in the TCGA-BRCA and METABRIC cohorts. (**A**,**E**) The xCell algorithm. (**B**,**F**) The quantiseq algorithm. (**C**,**G**) The ssGSEA algorithm. (**D**,**H**) MCPcounter algorithm.

We calculated the abundance of CD8+ T cells in TCGA-BRCA and METABRIC using four algorithms: MCP-counter, xCell, quantiseq, and ssGSEA. According to the median CD8+ T cell abundance, BC samples from both cohorts were classified into high and low groups. In the TCGA-BRCA, survival curves indicated that lower CD8+ T cell abundance was associated with a poorer prognosis (Figure 1A–D). Similarly, in the METABRIC cohort, BC patients with lower CD8+ T cell abundance had a worse prognosis (Figure 1E–I).

### 3.2. WGCNA Analysis Based on the CD8+ T Cell Abundance

**Figure 2:**
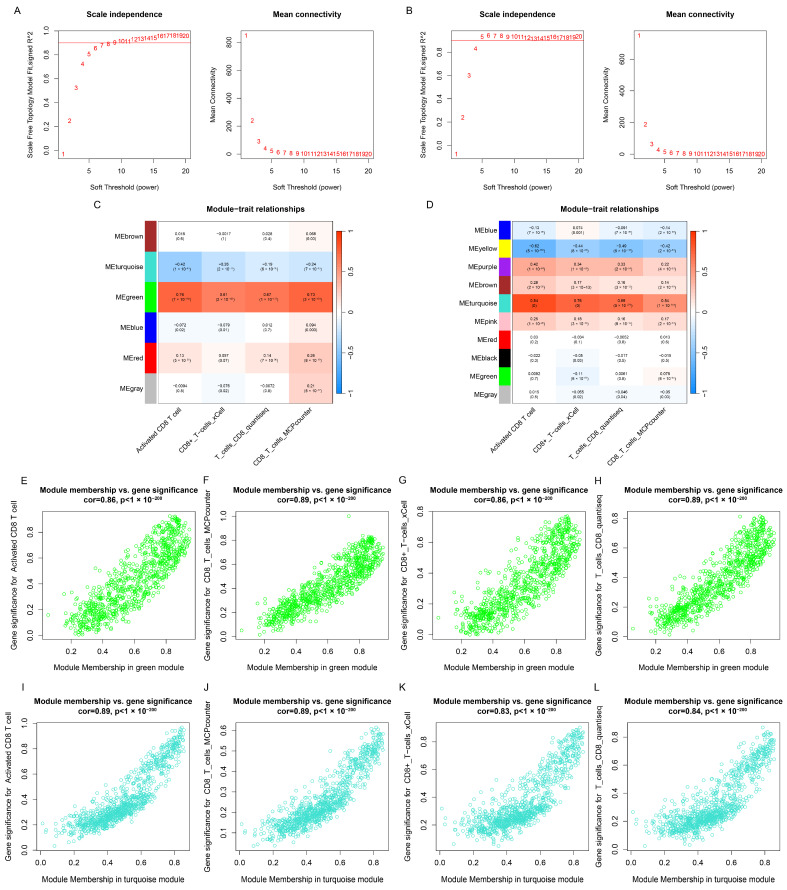
WGCNA analysis based on CD8+ T cell abundance. (**A**,**B**) Evaluation of the soft threshold in the TCGA-BRCA and METABRIC. (**C**,**D**) Correlation between each gene co-expression module and CD8+ T cell abundance in both datasets. Scatter plots of the green module MM and GS in the TCGA-BRCA cohort, including Activated_CD8_T_cell_ssGSEA (**E**), T_cells_CD8_MCPcounter (**F**), T_cells_CD8_xCell (**G**), and T_cells_CD8_quantiseq (**H**). Scatter plots of the turquoise module MM and GS in the METABRIC cohort, including Activated_CD8_T_cell_ssGSEA (**I**), T_cells_CD8_MCPcounter (**J**), T_cells_CD8_xCell (**K**), and T_cells_CD8_quantiseq (**L**).

The CD8+ T cell abundance of the four algorithms mentioned above were selected for WGCNA analysis in the TCGA-BRCA and METABRIC cohorts, respectively. The soft threshold power was six for TCGA-BRCA (Figure 2A) and four for METABRIC (Figure 2B). In the TCGA-BRCA cohort, system clustering identified six modules (Figure 2C), with the green module showing the highest correlation with Activated_CD8_T_cell_ssGSEA (Cor = 0.76), T_cells_CD8_MCPcounter (Cor = 0.61), T_cells_CD8_xCell (Cor = 0.67), and T_cells_CD8_quantiseq (Cor = 0.73). In the METABRIC cohort, ten modules were identified (Figure 2D), with the turquoise module showing the highest correlation. Within the green and turquoise modules, a positive correlation was found between module membership (MM) and gene significance (GS) (Figure 2E–L).

### 3.3. Identification and Enrichment Analysis of CTRGs

A Venn diagram revealed 147 intersecting genes between the green module, turquoise module, and DEGs (Figure S1A, Table S3). GO and KEGG enrichment analyses were undertaken to understand the biological processes of these genes. KEGG results showed that CTRGs were mainly enriched in cytokine–cytokine receptor interaction, cell adhesion molecules, and the TNF signaling pathway (Figure S1B). GO results indicated that CTRGs were mainly enriched in leukocyte cell–cell adhesion, regulation of T cell activation, regulation of cell–cell adhesion, and leukocyte-mediated immunity, among other immune-related biological processes (Figure S1C).

### 3.4. Screening for Prognostic-Related CTRGs

**Figure 3:**
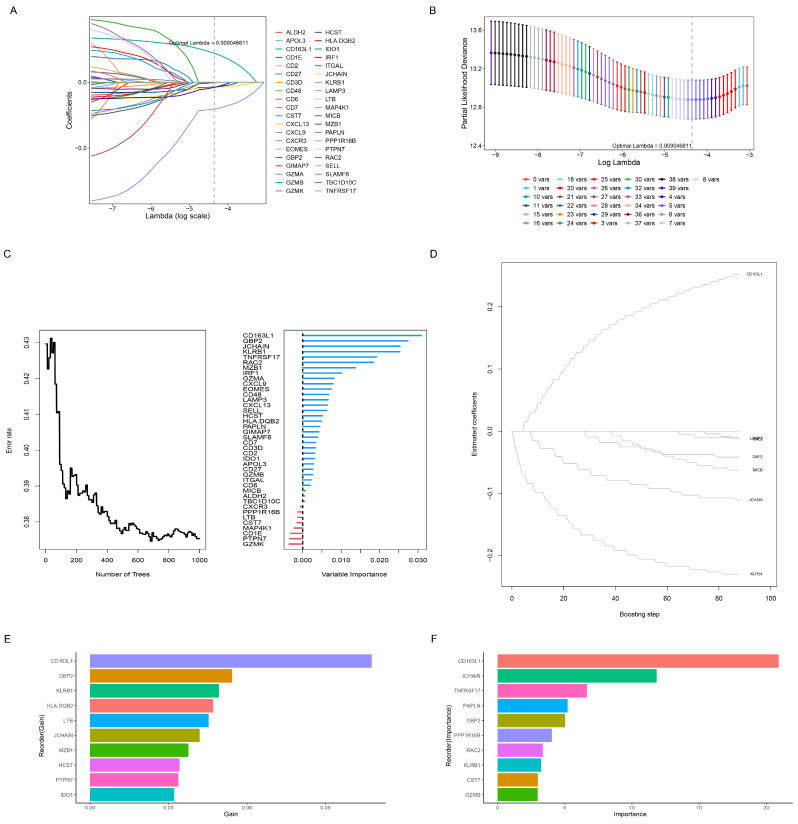
Screening of prognostic CTRGs through machine learning algorithm. (**A**) Plot of ten-fold cross-validations. (**B**) Plot of the LASSO coefficient. Screening of prognostic CTRGs using the Random Forest algorithm (**C**), and CoxBoost algorithm (**D**). (**E**) The top 10 most vital genes selected by the XGboost algorithm. (**F**) The top 10 most vital genes selected by the GBM algorithm.

**Figure 4:**
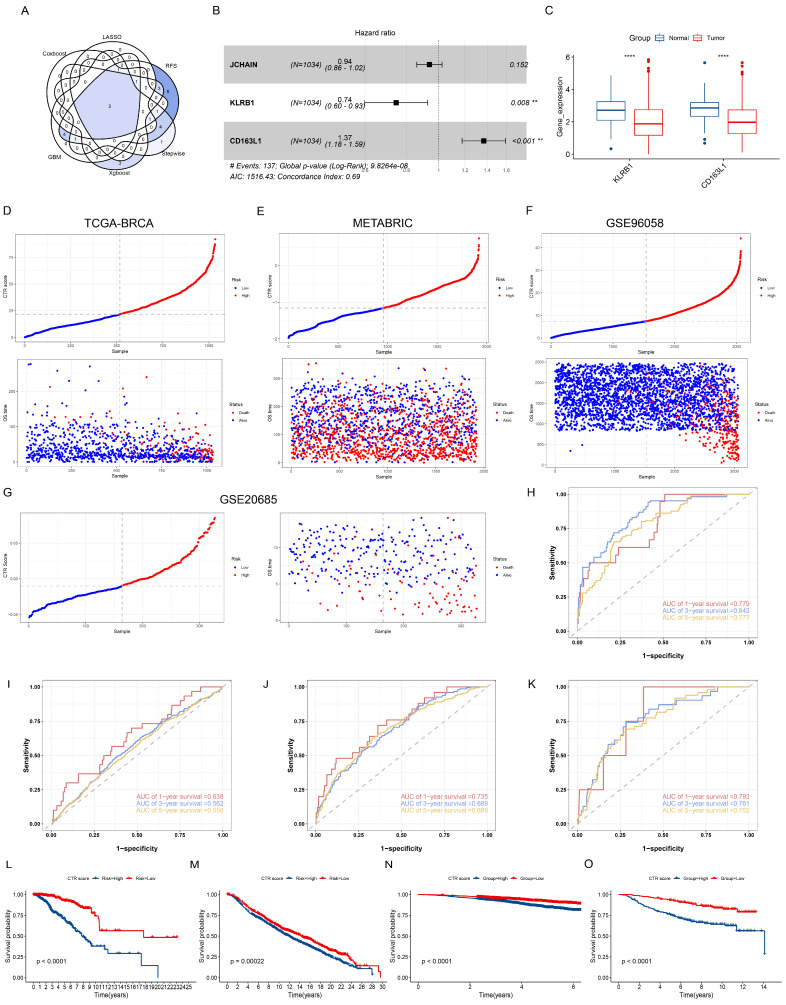
Construction of prognostic signature related to CD8+ T cells. (**A**) Venn diagram of six algorithms. (**B**) Identifying CTRGs with independent prognostic value. (**C**) Differential expression of two prognostic signature genes in normal and tumor tissues (**** *p* < 0.0001). Scatter plots of CTR score and survival status in the TCGA-BRCA (**D**), METABRIC (**E**), GSE96058 (**F**), and GSE20685 (**G**) datasets. (**H**–**K**) ROC curves of CTR score in the TCGA-BRCA, METABRIC, GSE96058, and GSE20685 datasets. Survival curves in the TCGA-BRCA (**L**), METABRIC (**M**), GSE96058 (**N**), and GSE20685 (**O**) datasets.

A total of 41 CTRGs were successfully screened by the one-factor Cox analysis (Figure S1D). Subsequently, seven machine learning algorithms were employed to further screen prognostic-associated CTRGs. The LASSO, CoxBoost, stepwise, and RFS algorithms identified six, seven, eight, and 27 genes, respectively (Figure 3A–E). Additionally, the top 10 most important genes in the XGboost and GBM algorithms were identified (Figure 3F,G). We further screened for the overlapping genes in the six algorithms by multivariate Cox analysis. Figure 4A shows the two intersecting genes from the six algorithms. The results of the multifactorial Cox showed that KLRB1 and CD163L1 had independent prognostic value (Figure 4B). Compared to normal tissue, the expression of KLRB1 and CD163L1 is lower in tumor tissue (Figure 4C).

### 3.5. Construction of Prognostic Signature Related to CD8+ T Cells

We build the CTR score by the GBM algorithm with the highest C-index (Figure S2). CTR score and survival statuses in the TCGA-train and TCGA-validation sets are shown in Figure S3A,B. Figure 4D–G show CTR score and survival statuses in the TCGA-BRCA, METABRIC, GSE96058 and GSE20685. In the TCGA-train set, the AUC values of the CTR score predicting the overall survival (OS) of BC patients at 1, 3, and 5 years were 0.785, 0.860, and 0.796 (Figure S3C). The AUC values in the TCGA-validation set were 0.599, 0.754 and 0.727. (Figure S3D). In the TCGA-all cohort, the AUC values were 0.779, 0.842, and 0.777 (Figure 4H). In the METABRIC cohort, the AUC values were 0.638, 0.562, and 0.550 (Figure 4I). In the GSE96058 cohort, the AUC values were 0.735, 0.689, and 0.689 (Figure 4G). In the GSE20685 cohort, the AUC values of were 0.793, 0.761, and 0.752 (Figure 4K). Survival curves indicate that in the TCGA-train, TCGA-validation, TCGA-all, METABRIC, and GSE96058 sets, BC patients in the high CTR score group have a worse prognosis (Figure 4L–O; Figure S3E,F).

### 3.6. Relationship between CTR Score and CD8+ T Cell Abundance

**Figure 5:**
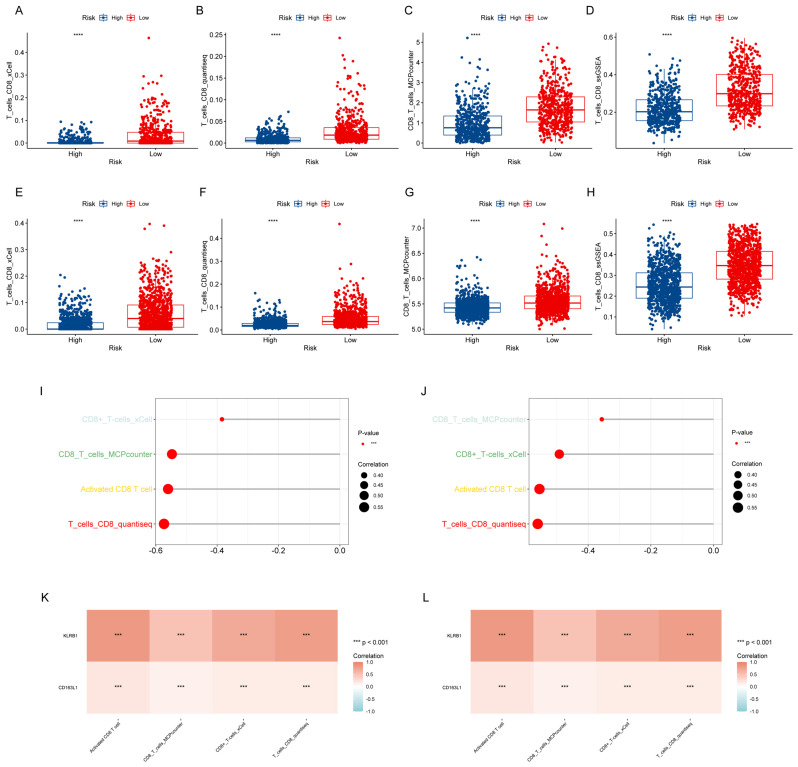
Association of CTR score with CD8+ T cell abundance. Comparison of CD8+ T cell abundance estimated by four algorithms in low and high CTR score groups in TCGA-BRCA (**A**–**D**) and METABRIC (**E**–**H**) (**** *p* < 0.0001). Assessment of the association between CTR score and CD8+ T cell abundance estimated by four algorithms in TCGA-BRCA (**I**) and METABRIC (**J**) (*** *p* < 0.001). Correlation analysis between two prognostic signature genes and CD8+ T cell abundance, estimated by four algorithms in TCGA-BRCA (**K**) and METABRIC (**L**).

We analyzed the relationship between CD8+ T cell levels, as assessed by the previous four algorithms, and the CTR score. The low CTR score group exhibited higher levels of CD8+ T cells in both TCGA-BRCA (Figure 5A–D) and METABRIC (Figure 5E–H). Correlation analyses revealed that the CTR score was negatively correlated with the levels of CD8+ T cells as determined by the four algorithms in TCGA-BRCA (Figure 5I) and METABRIC (Figure 5J) (*p* < 0.001). Additionally, we found that the expression levels of the two genes were positively correlated with the levels of CD8+ T cells by the four algorithms (Figure 5K–L).

### 3.7. Relationship between CTR Score and Clinical Characteristics

**Figure 6:**
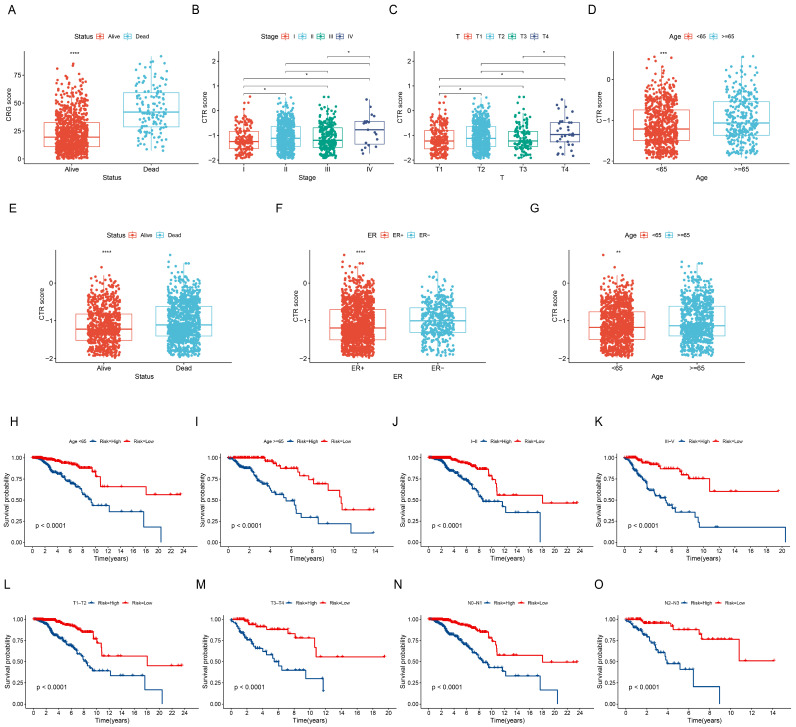
Relationship between CTR score and clinical characteristics. Differences in CTR score in the TCGA-BRCA cohort by status (**A**), stage (**B**), T stage (**C**), and age (**D**) (* *p*-value < 0.05; ** *p*-value < 0.01; *** *p*-value < 0.001; **** *p*-value < 0.0001). Differences in CTR score in the METABRIC cohort by status (**E**), ER (**F**), and age (**G**). Survival curves for different risk groups in the TCGA-BRCA cohort for ages < 65 (**H**), ages ≥ 65 (**I**), stages I–II (**J**), stages III–VI (**K**), T1–T2 (**L**), T3–T4 (**M**), N0–N1 (**N**), and N2–N3 (**O**).

To further estimate the performance of the CTR score, we analyzed its relationship with clinical features. Results revealed that in the TCGA-BRCA cohort, the CTR score showed significant differences across various statuses, stages, T stages, and age groups (Figure 6A–D). In the METABRIC cohort, significant differences in the risk score were observed across different statuses, estrogen receptor (ER) status, and age groups (Figure 6E–G). Next, we assessed the predictive ability of the prognostic feature across different clinical subgroups. The prognosis of BC patients in different CTR score groups showed significant differences among subgroups under 65 years old, 65 years and older, stages I–II, stages III–IV, T1–T2, T3–T4, N0–N1, and N2–N3 (Figure 6H–O). In addition, we analyzed the performance of CTR score in patients with different subtypes of breast cancer in the TCGA-BRCA and GSE96058 cohorts. Notably, CTR score showed robust predictive performance across all subtypes (Figure S4A–D).

### 3.8. Construction of a Nomogram

**Figure 7:**
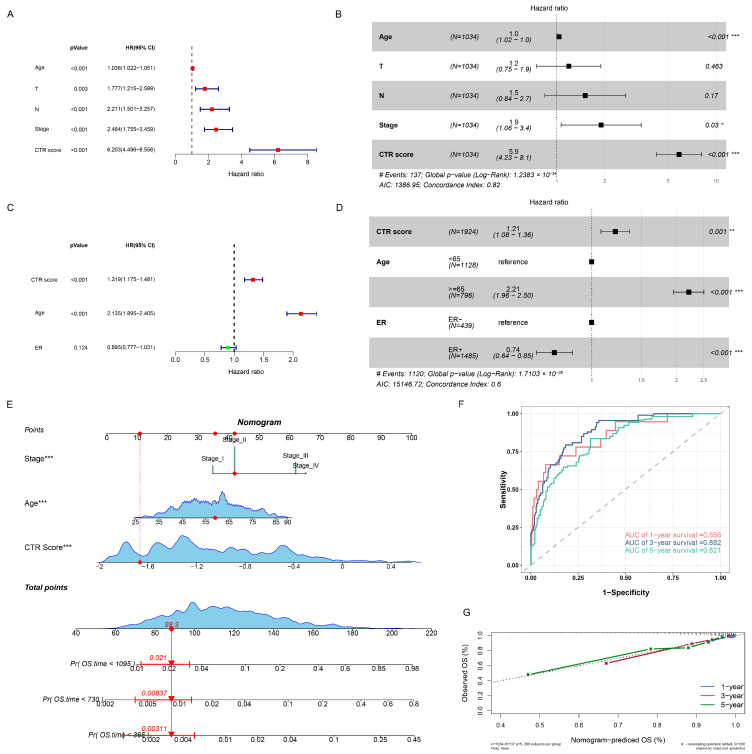
Construction of a nomogram. Assessment of the independence of the CTR score in the TCGA-BRCA (**A**,**B**) and METABRIC (**C**,**D**) cohorts (* *p*-value < 0.05; ** *p*-value < 0.01; *** *p*-value < 0.001). (**E**) Nomogram in the TCGA-BRCA. (**F**) ROC curve of the nomogram. (**G**) Calibration curve of the nomogram.

Univariate and multivariate Cox analyses of CTR score and clinical characteristics were applied aiming to explore the independence of CTR scores. The results demonstrated that the CTR score served as an independent predictive indicator separate from other clinical features in the TCGA-BRCA, METABRIC, GSE96058, and GSE20685 (Figure 7A–D; Figure S5A–D). In the TCGA-BRCA cohort, a nomogram was constructed combining age and stage that could predict the 1-, 3-, and 5-year survival of BC patients (Figure 7E). The ROC curves indicated that the AUC values of the nomogram for predicting 1-, 3-, and 5-year survival were 0.856, 0.882, and 0.821 (Figure 7F). Besides, the survival probabilities predicted by the nomogram matched the actual survival probabilities substantially (Figure 7G).

### 3.9. Differential Biological Processes between Different CTR Score Groups

The GSVA-GO analysis revealed that the high CTR score group exhibited enrichment in metabolism-related biological processes, such as nucleoside phosphate metabolic process, thioester metabolic process, and fatty acyl CoA synthase activity. Conversely, the low CTR score group demonstrated enrichment in immune-related biological processes, including lymphocyte anergy, positive regulation of T cell receptor signaling pathway, and CD40 signaling pathway (Figure S6A). Similarly, GSVA-KEGG analysis indicated that the high CTR score group exhibited enrichment in butanoate metabolism, histidine metabolism, and phenylalanine metabolism, while the low-risk group was in the ERBB signaling pathway, leukocyte transendothelial migration, and tight junction (Figure S6B). In addition, we investigated differences in cancer-related biological processes across CTR score groups. The low CTR score group displayed higher scores in angiogenesis, apoptosis, proliferation, differentiation, and inflammation, whereas the high-risk group showed elevated scores in cell cycle, DNA repair, and glycolysis (Figure S6C–J).

### 3.10. Relationship between CTR Score and Immune Cell Infiltration

**Figure 8:**
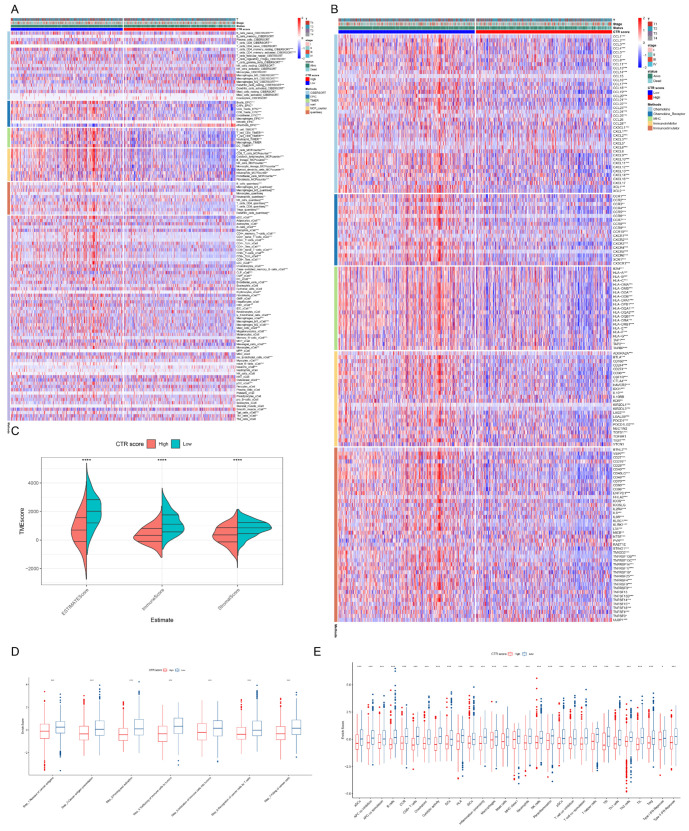
Relationship between CTR score and immune cell infiltration. (**A**) Differences in immune infiltration levels between different CTR score groups assessed by five algorithms. (**B**) Differences in tumor microenvironment scores (immune, stromal, and estimate score) between CTR score groups (**** *p* < 0.0001). (**C**–**E**) Association of CTR score with immune checkpoints, cancer immune cycles and 29 immune characteristics (* *p* < 0.05, ** *p* < 0.01, *** *p* < 0.001, **** *p* < 0.0001).

As depicted in Figure 8A,B, the low CTR score group exhibited elevated levels of immune cells and demonstrated higher immune, stromal, and estimate scores. Moreover, significant differences were observed in immunoinhibitor, immunostimulator, MHC, chemokine, and chemokine receptor expression between the CTR score groups (Figure 8C). Furthermore, our analysis revealed that the cancer immune cycle score and 29 immune characteristics score were notably higher in the low CTR score group (Figure 8D,E).

### 3.11. Prediction of Immune Therapy and Chemotherapy Sensitivity

**Figure 9:**
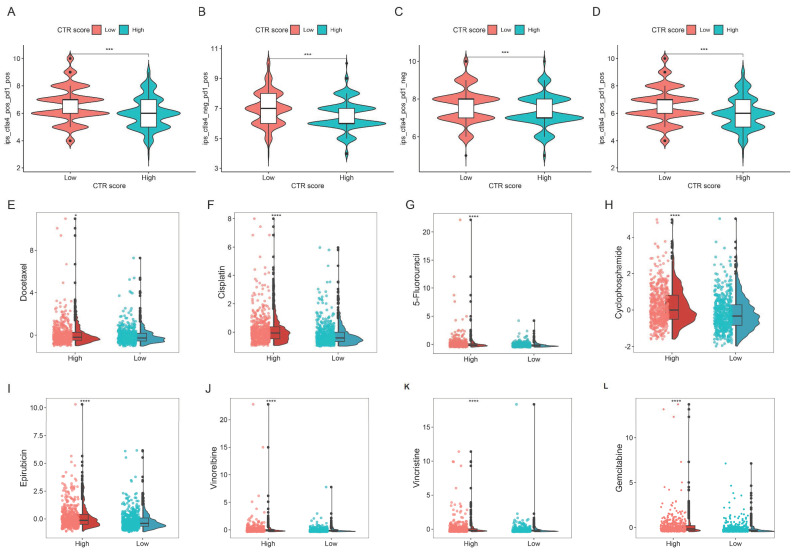
Differences in sensitivity to immunotherapy and chemotherapeutic agents in various CTR score groups. (**A**–**D**) Differences in Immune Phenotype Scores (IPS) between different groups (*** *p* < 0.001). (**E**–**L**) Differences in chemotherapy sensitivity to docetaxel, cisplatin, 5-fluorouracil, cyclophosphamide, epirubicin, vinorelbine, vincristine, and gemcitabine between different groups (* *p* < 0.05, **** *p* < 0.0001).

The Immune Phenotype Score (IPS) is a predictive factor for the response to CTLA-4 and PD-1 antibodies [21]. As depicted in Figure 9A–D, the IPS was higher in the low CTR score group, suggesting a better response to immune checkpoint inhibitors. We also analyzed the sensitivity to chemotherapy-related drugs across different CTR score groups. The low CTR score group had better sensitivity to docetaxel, cisplatin, 5-fluorouracil, cyclophosphamide, epirubicin, vinorelbine, vincristine, and gemcitabine (Figure 9E–L).

### 3.12. Single-Cell Analysis of Prognostic Signature Genes

**Figure 10:**
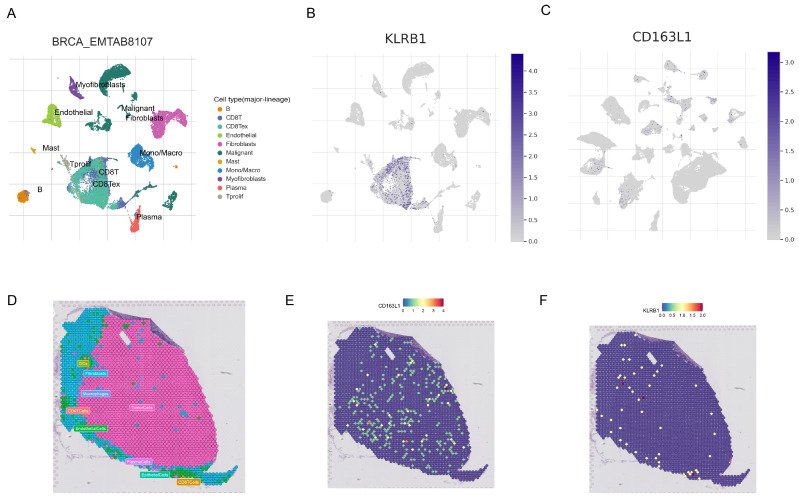
Single-cell and spatial transcriptomic analysis of prognostic feature genes. (**A**) Different cell types in the EMTAB8107 dataset. Expression levels of KLRB1 (**B**) and CD163L1 (**C**) in different cells. (**D**) Distribution of different cells in GSE203612-GSM6177603-NYU-BRCA2. (**E**,**F**) Distribution of CD163L1 and KLRB1 in different cells.

Figure 10A displays various cell types in the EMTAB8107 dataset. Single-cell analysis revealed that KLRB1 is mainly enriched in CD8+ T cells, while CD163L1 is expressed at lower levels across different cells (Figure 10B,C). Figure 10D shows the distribution of different cells in GSE203612-GSM6177603-NYU-BRCA2. CD163L1 is abundantly expressed in malignant cells, whereas KLRB1 shows lower expression across various cells (Figure 10D,E).

### 3.13. Validation of Expression Levels of Prognostic Signature Genes

**Figure 11:**
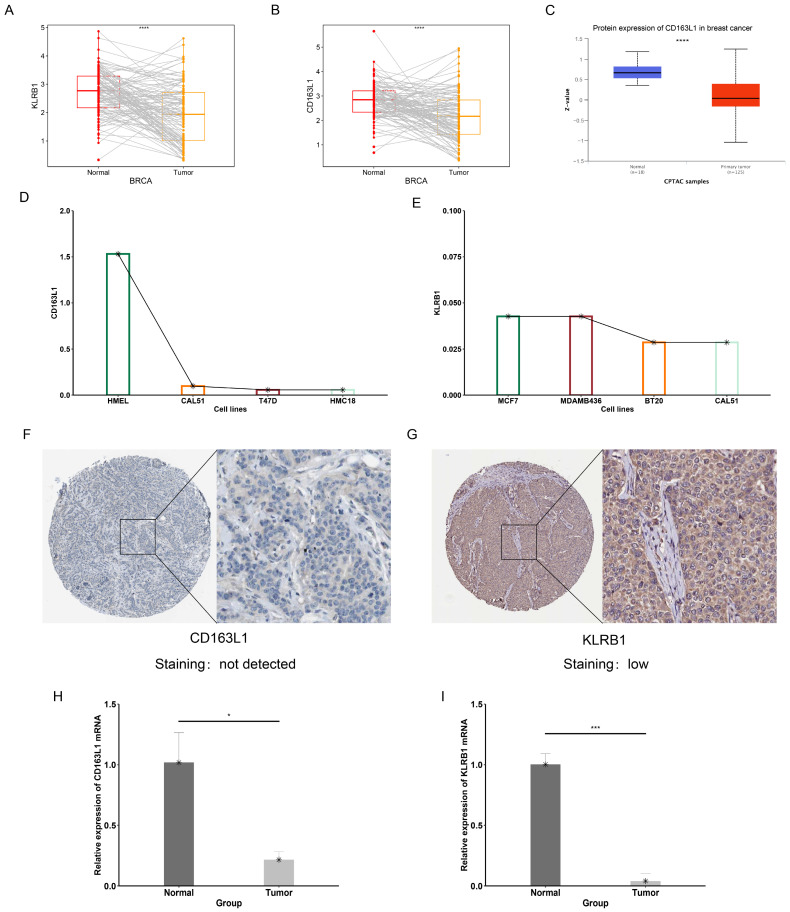
Validation of expression levels of prognostic-related CTRGs. (**A**,**B**) Expression levels of KLRB1 and CD163L1 in cancerous tissues and paired adjacent non-cancerous tissues (**** *p* < 0.0001). (**C**) Expression of CD163L1 protein in the UALCAN database (**** *p* < 0.0001). (**D**) Expression levels of CD163L1 in normal epithelial cell line (HMEL) and breast cancer cell lines (CAL51, T47D, and HMC18) in the CCLE database. (**E**) Expression level of KLRB1 in breast cancer cell lines. (**F**,**G**) IHC staining images of KLRB1 and CD163L1 proteins in cancerous tissues. (**H**,**I**) Experimental validation of the prognostic CTRGs (* *p* < 0.05, *** *p* < 0.001).

Compared to paired normal tissues, the expression levels of KLRB1 and CD163L1 were reduced in cancer tissues (Figure 11A,B). Consistent with previous results, the protein expression level of CD163L1 is also downregulated in cancer tissues (Figure 11C). Figure 11D,E, show the expression levels of KLRB1 and CD163L1 in breast cancer cell lines. Immunohistochemistry (IHC) images of the prognostic feature genes in tumor tissues were downloaded from the Human Protein Atlas (HPA) database. As shown in Figure 11F,G, the staining of KLRB1 and CD163L1 proteins is lighter in tumor tissues. The expression of KLRB1 and CD163L1 mRNA is downregulated in tumor tissues compared to paracancerous tissue (Figure 11H,I).

## 4. Discussion

Over the past 50 years, the mortality rate for breast cancer has decreased by 58% due to advancements in early screening techniques and treatment methods [22,23]. However, despite these improvements, the incidence of breast cancer remains on the rise [24,25]. Breast cancer encompasses various biological entities, each characterized by specific genomic alterations, gene expression patterns, and influences from the TME, all of which collectively determine clinical behaviors and treatment responses [26]. The TME itself is a complex ecosystem, comprising cancer cells, various non-cancerous cells, and an altered, vascularized extracellular matrix [27]. It includes immune cells (such as T lymphocytes, B lymphocytes, natural killer cells, mast cells, and neutrophils), stromal cells, extracellular matrix, and secretory molecules [28]. The interactions among these diverse cells can either promote or inhibit the progression of tumors, influencing the developmental trajectory of the TME [29]. Given the complexity of the TME, a variety of therapeutic strategies targeting it have been developed, such as immunotherapies, anti-angiogenic drugs, and treatments that target tumor-promoting cells [30].

As a protective component of immune cells within the TME, cytotoxic CD8+ T cells are critical in the elimination of malignant cells [31]. Although these cells are present in tumor tissues, they often find themselves in an exhausted state within cancerous environments, which impairs their anti-tumor activities [32,33]. Additionally, CD8+ T cells work in concert with other cells within the TME to exert anti-tumor effects. For instance, FOLR2+ macrophages have been shown to interact with CD8+ T cells to enhance the anti-tumor immune response [34]. Recent studies have underscored the importance of tumor-resident type 1 conventional dendritic cells (cDC1) in orchestrating CD8+ T cell-mediated anti-cancer responses at the tumor-intrinsic stage [35]. In cancers such as gastric, lung, colon, and ovarian, higher levels of CD8+ T cell infiltration have correlated with improved clinical outcomes [36,37,38,39]. Moreover, studies have highlighted that tumor-resident memory CD8+ T cells with a structured residency phenotype are associated with favorable prognoses in patients with triple-negative breast cancer and play significant roles in the efficacy of checkpoint blockade therapies and sustaining protective immunity against breast cancer [40]. In this study, we also observed that breast cancer patients with higher levels of CD8+ T cell infiltration exhibited better prognoses. Although CD8+ T cells are crucial for anti-tumor immunity, their specific mechanisms of action in the TME still require further investigation.

Given the pivotal role of CD8+ T cells in the tumor microenvironment (TME), we developed the CTR score based on these cells using machine learning algorithms. We found that breast cancer patients categorized in the high CTR score group exhibited a poorer prognosis and had reduced infiltration of CD8+ T cells. The CTR score is an independent prognostic predictor for BC patients and demonstrated robust predictive performance in different datasets. In addition, we found that CTR score could well predict the prognosis of BC patients with different clinicopathological features. These results suggest that CTR score can serve as a potential biomarker for predicting the prognosis of BC patients. To enhance clinical utility, we integrated the CTR score with clinical pathological characteristics to construct a nomogram designed to predict the prognosis of breast cancer patients. Both calibration curves and ROC curves demonstrate that this nomogram provides good predictive accuracy.

The CTR score incorporates two genes: Killer Cell Lectin Like Receptor B1 (KLRB1) and CD163 Molecule Like 1 (CD163L1). Prior research has established a strong link between these genes and cancer progression. For instance, high expression of KLRB1 has been shown to promote the progression and evolution of gliomas by influencing T cell dysfunction [41]. Another study revealed that inactivation of the KLRB1 gene or antibody-mediated blockade of CD161 can enhance T cell-mediated anti-tumor functions in glioblastoma [42]. In our analysis, KLRB1 was found to be downregulated in breast cancer while being abundantly expressed in CD8+ T cells. As for CD163L1, which is a homolog co-localized with CD163, it is typically highly expressed in macrophages and associated with tissue-resident macrophages that exhibit anti-inflammatory or immunologically incompetent phenotypes, potentially playing a role in the resolution of inflammation [43,44]. In our study, we observed that CD163L1 is downregulated in breast cancer and closely linked to CD8+ T cell dynamics. Furthermore, RT-PCR validation of these genes corroborated our database analyses, thereby reinforcing the predictive accuracy of our prognostic signature.

The complex interactions between immune cells and cancer cells within the TME are considered key mechanisms regulating tumor progression [45]. In this study, we found that the levels of B cells, CD8+ T cells, and M1 macrophages were higher in the low-risk group. Previous studies have demonstrated that these immune cells play a significant role in inhibiting cancer progression [46,47,48]. Conversely, the infiltration of M0 and M2 macrophages was higher in the high-risk group. M0 and M2 macrophages have also been shown to promote tumor development [49,50,51]. Furthermore, we observed that the scores of cancer immune cycles were higher in the low CTR score group, indicating a heightened level of anti-cancer immune activity within this group. These results suggest that TME in the high CTR score group is strongly associated with immunosuppression, which may be the cause of the poorer prognosis in this group.

Recent research emphasizes the importance of a multidisciplinary approach in decision-making for breast cancer treatment, particularly in selecting systemic therapies tailored to individual patients [52]. The primary local treatment for breast cancer remains surgical intervention, supplemented by adjuvant therapies such as chemotherapy, radiotherapy, and endocrine therapy [53]. Our study also revealed that the low CTR score group demonstrated lower IC50 values for chemotherapy drugs, suggesting a more favorable response to these treatments. Furthermore, the last decade has seen significant advancements in immunotherapy, which has revitalized cancer treatment protocols. Various immunotherapeutic approaches, including immune checkpoint inhibitors, vaccination, and adoptive cell transfer, have been rigorously tested in clinical settings, especially for patients with triple-negative breast cancer (TNBC) [54]. Exhausted T cells, which are prevalent in the TME, typically overexpress various inhibitory surface molecules that hinder T cell activation, such as CTLA-4 and PD-1 [55,56]. These molecules are targeted by immune checkpoint inhibitors, which aim to disrupt regulatory pathways in T cells and enhance anti-tumor immune responses [57]. In this context, our findings indicate that the low CTR score group exhibited higher IPS, suggesting a more effective response to anti-PD-1 and anti-CTLA-4 therapies. These results further underscore the potential of using the CTR score as biomarker for predicting clinical treatment responses in breast cancer.

Certainly, there are some limitations in this study that should be further addressed. Firstly, our prognostic feature was constructed based on databases and still needs further validation in real cohorts. Moreover, the role and potential molecular mechanisms of the two prognostic feature genes in the progression of breast cancer need to be further explored in in vitro or in vivo experiments.

## 5. Conclusions

In conclusion, we established a CD8+ T cell-related prognostic signature in BC by machine learning, which can be used as an independent prognostic factor for BC. In addition, the prognostic signature can assess the tumor microenvironment as well as the response to immunotherapy and chemotherapy in BC patients. These results provide an important reference for clinical outcome monitoring as well as early treatment of breast cancer patients.

## Data Availability

The datasets used in this study can be found in The Cancer Genome Atlas database (https://portal.gdc.cancer.gov/, accessed on 8 January 2024), cBioPortal for Cancer Genomics (https://www.cbioportal.org/, accessed on 8 February 2024), and Gene Expression Omnibus (https://www.ncbi.nlm.nih.gov/geo/, accessed on 15 February 2024).

## Supplementary Materials

The following supporting information can be downloaded at: https://www.mdpi.com/article/10.3390/genes15081093/s1, Figure S1: Identification and enrichment analysis of CTRGs; Figure S2: Constructing the prognostic signature through machine learning algorithms; Figure S3: Construction of prognostic signatures related to CD8+ T cells; Figure S4: Relationship between CTR score and different subtypes; Figure S5: Investigate the independence of the CTR score; Figure S6: Differences in biological processes between different CTR score groups; Table S1: Comparison of baseline characteristics; Table S2: Primer sequences; Table S3: Intersection genes between green module, turquoise module and differentially expressed genes.
