# Supplementary material for: Integration of Bioinformatics and Machine Learning to Identify CD8+ T Cell-Related Prognostic Signature to Predict Clinical Outcomes and Treatment Response in Breast Cancer Patients
Source: Genes (Basel). 2024 Aug 19;15(8):1093. doi: 10.3390/genes15081093 (PMC11353403; doi:10.3390/genes15081093)
Supplement: Supplementary file 1 [file genes-15-01093-s001.zip › genes-3139215-supplementary.pdf]

## Supplementary Figures

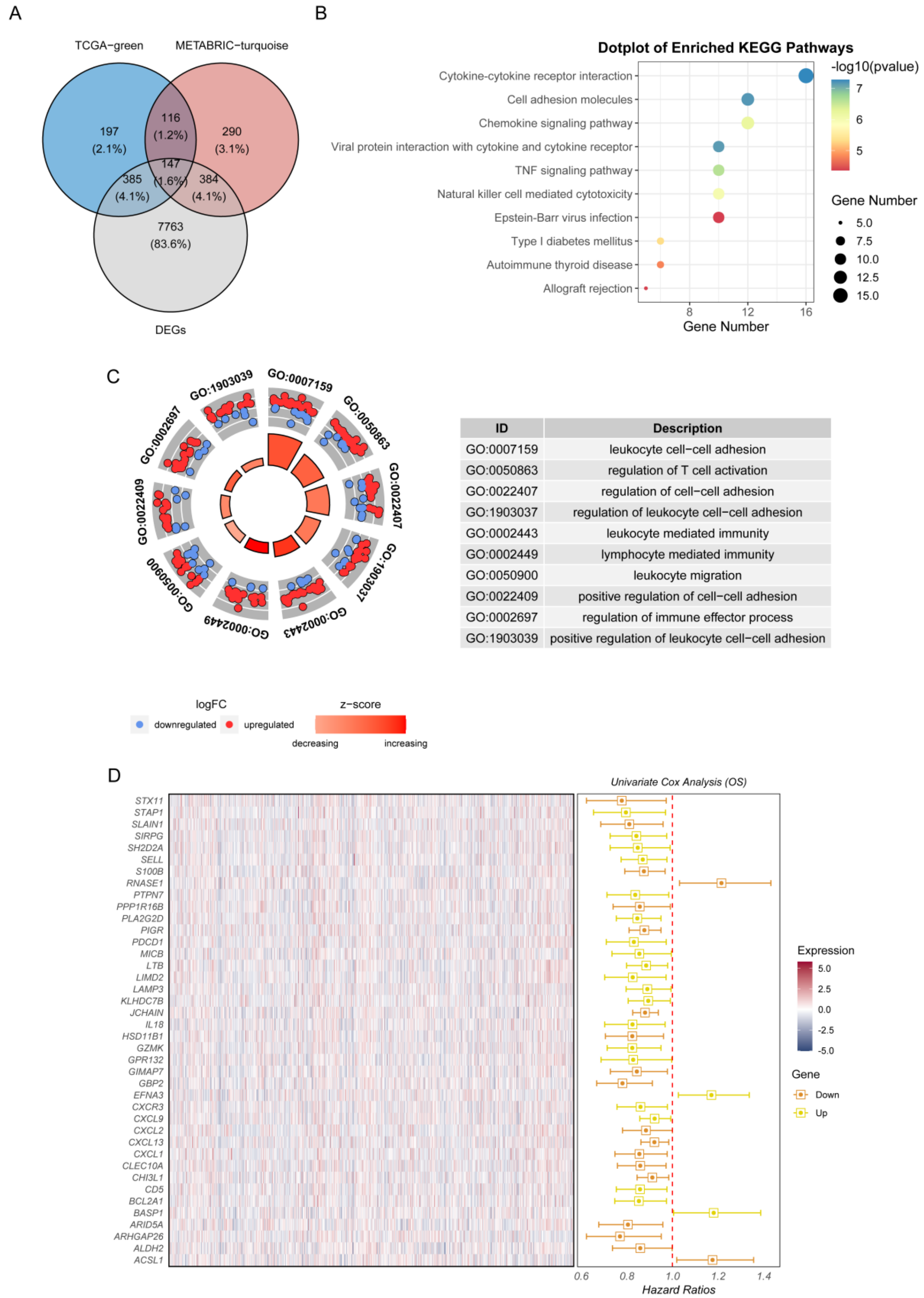


**Figure S1**. Identification and enrichment analysis of CTRGs. (**A**) Venn diagram of TCGA-green, METABRIC-turquoise, and DEGs. (**B**) GO analysis and (**C**) KEGG analysis of 147 CTRGs. (**D**) Univariate Cox analysis.

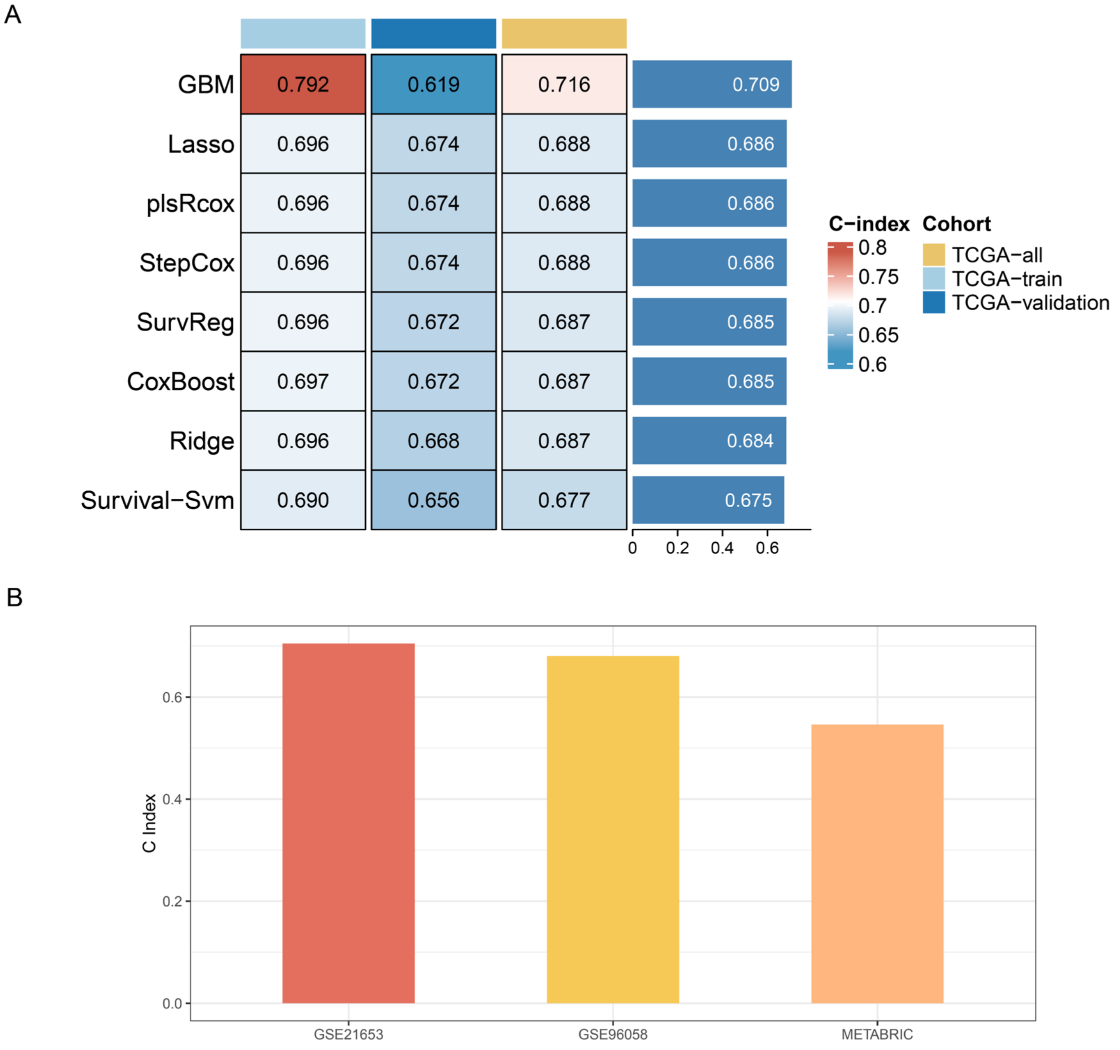


**Figure S2**. Constructing the CTR score through machine learning algorithms. (**A**) GBM was selected as the modeling method among10 machine learning algorithms for scoring. (**B**) C-index of the CTR score in the validation datasets.

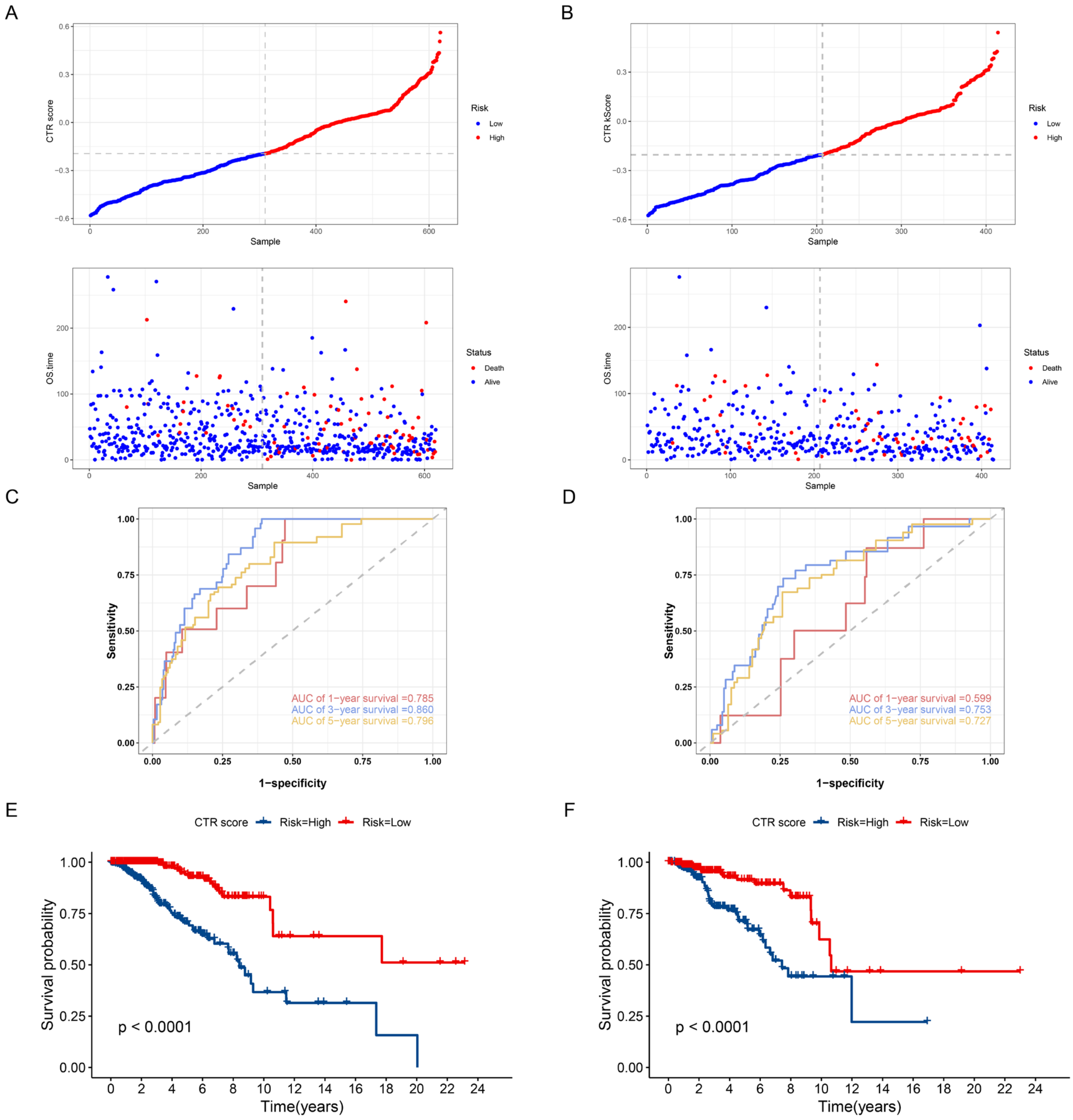


**Figure S3**. Construction of prognostic signature related to CD8+T cells. CTR score in the TCGA-train and TCGA-validation sets and scatter plots of survival status (**A-B**). ROC curves for predicting 1, 3, and 5-year survival rates using the CTR score in the TCGA-train (**C**) and TCGA-validation (**D**) sets. Survival curves for high and low CTR score groups in the TCGA-train (**E**) and TCGA-validation (**F**) sets.

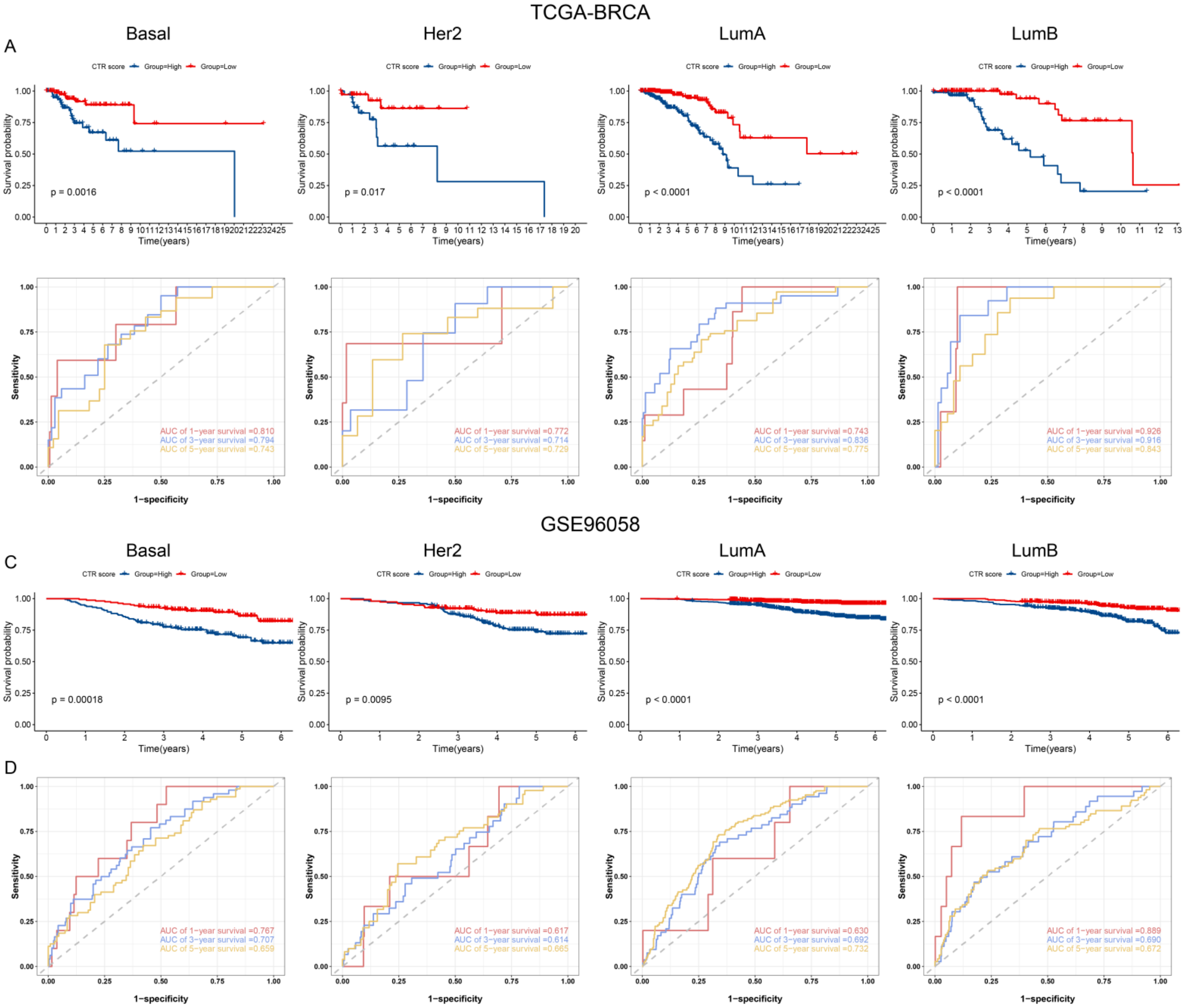


**Figure S4**. Relationship between CTR score and different subtypes. (**A**) Survival curves for different CTR score groups in the TCGA-BRCA cohort for Basal, Her2, LumA, and LumB. (**B**) ROC curves for predicting 1, 3, and 5-year survival rates using the CTR score in the TCGA-BRCA dataset. (**C**) Survival curves for different CTR score groups in the GSE96058 cohort for Basal, Her2, LumA, and LumB. (**D**) ROC curves for predicting 1, 3, and 5-year survival rates using the CTR score in the GSE96058 dataset.

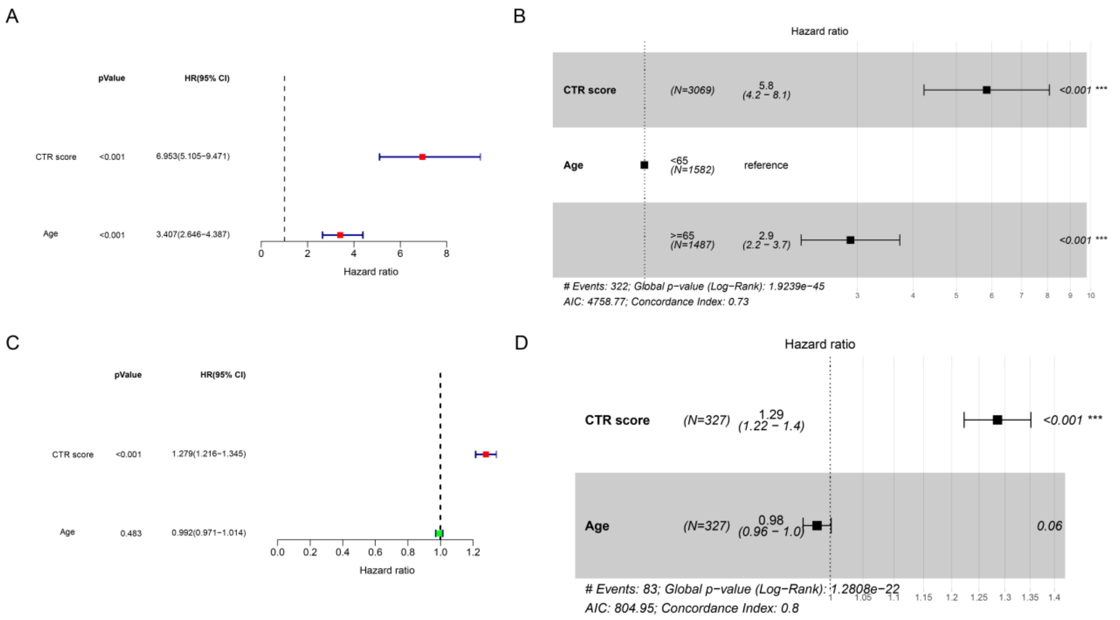


**Figure S5**. Investigate the independence of the CTR score. Univariate Cox regression analysis and multivariate Cox regression analysis assess the independence of the CTR score in the GSE96058 (**A**, **B**) and GSE20685 (**C**, **D**) cohorts.

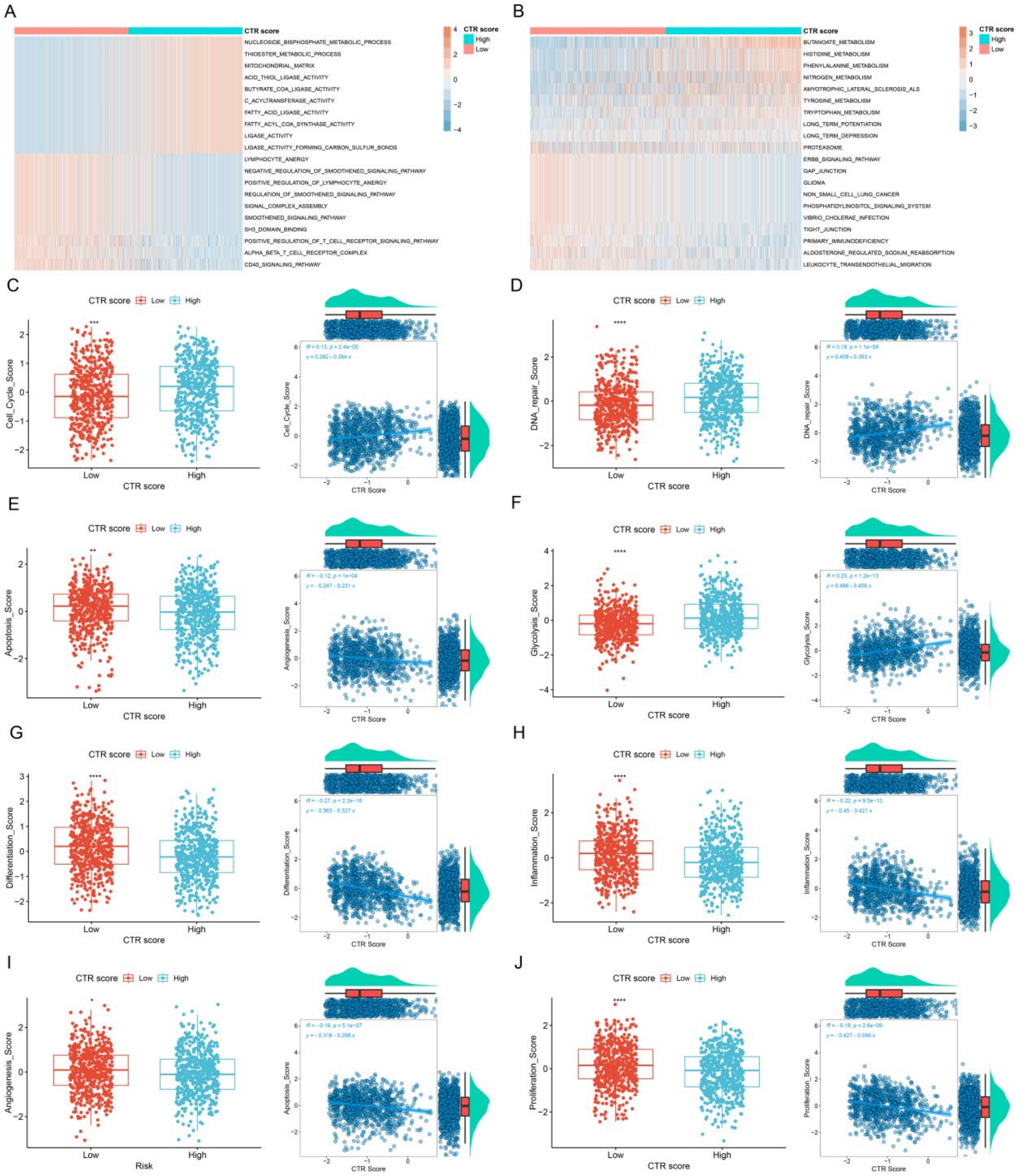


**Figure S6**. Differences in biological processes between different CTR score groups. (**A**, **B**) GSVA analysis of differentially expressed genes among CTR score groups. (**C-J**) Correlation between CTR score and cell cycle score, DNA repair score, apoptosis score, glycolysis score, differentiation score, inflammation score, angiogenesis score, and proliferation score.
